# Pravastatin-induced improvement in coronary reactivity and circulating ATP and ADP levels in young adults with type 1 diabetes

**DOI:** 10.3389/fphys.2012.00338

**Published:** 2012-08-23

**Authors:** Tuomas O. Kiviniemi, Gennady G. Yegutkin, Jyri O. Toikka, Subhadeep Paul, Tero Aittokallio, Tuula Janatuinen, Juhani Knuuti, Tapani Rönnemaa, Juha W. Koskenvuo, Jaakko J. Hartiala, Sirpa Jalkanen, Olli T. Raitakari

**Affiliations:** ^1^Department of Clinical Physiology, Turku University HospitalTurku, Finland; ^2^Department of Medicine, Turku University HospitalTurku, Finland; ^3^Medicity Research Laboratory, University of TurkuTurku, Finland; ^4^Department of MathematicsIIT Kharagpur, India; ^5^Institute for Molecular Medicine, University of HelsinkiHelsinki, Finland; ^6^Turku PET Centre, Turku University HospitalTurku, Finland; ^7^Research Centre of Applied and Preventive Cardiovascular Medicine, University of TurkuTurku, Finland

**Keywords:** ATP, ADP, soluble nucleotidases, coronary flow, pleiotropic effect

## Abstract

**Aims:** Extracellular ATP and ADP regulate diverse inflammatory, prothrombotic and vasoactive responses in the vasculature. Statins have been shown to modulate their signaling pathways *in vitro*. We hypothesized that altered intravascular nucleotide turnover modulates vasodilation in patients with type 1 diabetes (T1DM), and this can be partly restored with pravastatin therapy. **Methods:** In this randomized double blind study, plasma ATP and ADP levels and echocardiography-derived coronary flow velocity response to cold pressor test (CPT) were concurrently assessed in 42 normocholesterolemic patients with T1DM (age 30 ± 6 years, LDL cholesterol 2.5 ± 0.6 mmol/L) before and after four-month treatment with pravastatin 40 mg/day or placebo (*n* = 22 and *n* = 20, respectively), and in 41 healthy control subjects. **Results:** Compared to controls, T1DM patients had significantly higher concentrations of ATP (*p* < 0.01) and ADP (*p* < 0.01) and these levels were partly restored after treatment with pravastatin (*p* = 0.002 and *p* = 0.007, respectively), but not after placebo (*p* = 0.06 and *p* = 0.14, respectively). Coronary flow velocity acceleration was significantly lower in T1DM patients compared to control subjects, and it increased from pre- to post-intervention in the pravastatin (*p* = 0.02), but not in placebo group (*p* = 0.15). **Conclusions:** Pravastatin treatment significantly reduces circulating ATP and ADP levels of T1DM patients, and concurrently improves coronary flow response to CPT. This study provides a novel insight in purinergic mechanisms involved in pleiotropic effects of pravastatin.

## Introduction

Extracellular ATP and ADP are important signaling molecules regulating diverse signaling responses in cardiovascular, nervous and other systems (Ralevic and Burnstock, 1998; Bours et al., 2006; Erlinge and Burnstock, 2008). Released into extracellular fluids by exocytosis from nucleotide-containing granules, by efflux through a membrane transport system in response to cell activation, or as a consequence of cell death, they act as paracrine or autocrine mediators of inflammation (Bours et al., 2006). Moreover, they cause vasodilation via selective activation of a series of G-protein-coupled (P2Y) or ligand-gated (P2X) receptors (Ralevic and Burnstock, 1998; Erlinge and Burnstock, 2008; Mercier et al., 2012). ATP may stimulate vascular smooth muscle cell (VSMC) growth, migration, release of matrix metalloproteinases and osteopontin that may contribute to the development of diabetic microvascular disease (Erlinge and Burnstock, 2008). Abnormal reactive hyperemia as assessed with flow-mediated dilation (FMD) of brachial artery (Järvisalo et al., 2004) or abnormal coronary microcirculatory reactivity (Pitkänen et al., 1998) exists in patients with type 1 diabetes (T1DM) before any clinical signs or symptoms of macrovascular disease. The role of purinergic signaling cascade in the regulation of arterial vasodilation in the early stages of atherosclerosis of patients T1DM, however, remains largely unknown.

Statins activate the phosphoinositol-3 kinase and Akt signaling pathway, and in addition, increase expression and activity of endothelial enzymes such as nitric oxide synthase, cyclooxygenase-2 and ecto-5′-nucleotidase (Laufs et al., 1998; Ledoux et al., 2002; Merla et al., 2007). Ecto-5′-nucleotidase mediates rapid dephosphorylation of ATP/ADP-derived AMP further to adenosine, which has vasodilatory, anti-inflammatory (Bours et al., 2006) as well as cardioprotective responses against hypoxia (Eltzschig et al., 2003). The ability of atorvastatin to up-regulate ATPase, ADPase and ecto-5′-nucleotidase (AMPase) activities in human valve interstitial cells (Osman et al., 2006) suggests that the whole purinergic cascade could be modulated under the action of statins.

The aims of this trial were (1) to perform comprehensive comparative analysis of circulating ATP and ADP levels, soluble nucleotidase activities, coronary flow responses to CPT in control healthy subjects vs young asymptomatic, normocholesterolemic patients with non-complicated T1DM, and (2) to assess whether these biochemical and hemodynamic parameters would be concurrently modulated in T1DM patients in a prospective randomized placebo controlled four-month treatment with pravastatin.

## Materials and methods

### Patient selection and study design

Forty-two patients with non-complicated T1DM participated in the study. The inclusion criteria were: age 18–40 years, diabetes duration 3–25 years, no symptoms or diagnosis of cardiovascular disease or asthma, no use of cardiovascular medication or antioxidants, no diagnosis of proliferative retinopathy, FMD of the brachial artery less than 10%, HbA_1c_ less than 10%, LDL cholesterol less than 4.0 mmol/L and normal liver function. Altogether 57 patients were screened for the study. The patients were recruited by advertisement and from diabetes clinics in Turku and surrounding areas. The patients were interviewed for medical history, alcohol and caffeine use, physical activity and family history of premature coronary artery disease. In addition, serum human chorionic gonadotropin level was measured from all female patients to exclude pregnancy. The subjects were randomized in a double blind manner to receive either pravastatin 40 mg/day (*n* = 22) or placebo (*n* = 20) for four months. Bristol-Myers Squibb, Finland, provided Pravastatin 40 mg tablets and matching placebos. In order to control for use of dietary fats, the use of bread spread was standardized by providing all subjects margarine (total fat content 60 g/100 g whereof 18 g saturated, 29 g monounsaturated and 13 g polyunsaturated, vitamin E 12 mg/100 g, vitamin A 900 μg/100 g and vitamin D 7.5 μg/100 g) and advising to use 20 g/day of this product on bread. Otherwise the subjects were advised to adhere to their normal diet. The control group consisted of 41 healthy (mean age 24 ± 2.3 years) normocholesterolemic, non-hypertensive, and non-smoking Caucasian men.

The Local Ethical Committee of the Turku University Hospital approved the study protocol. The study was conducted according to the principles expressed in the Declaration of Helsinki. The study protocol and the potential risks of the study were explained in detail to the patients and thereafter a written informed consent was obtained.

Blood samples were taken after an overnight fast on study mornings from the antecubital vein. Blood was collected into tubes for preparing plasma and serum blood samples, respectively. For serum preparation, blood was allowed to clot before centrifugation (10 min at 1500 g) while plasma samples (EDTA) were immediately centrifuged and freezed at −80°C. Serum lipid levels, HbA1c were determined as previously reported (Janatuinen et al., 2004).

### Quantification of ATP and ADP levels in human plasma

Plasma ATP and ADP were determined by enzyme-coupled assay using ATPlite assay kit with a long-lived luminescent signal (Perkin Elmer, Groningen, The Netherlands) as described elsewhere (Mercier et al., 2012). Briefly, 10-μl aliquots of EDTA-plasma from healthy as well as placebo and pravastatin-treated T1DM were transferred into two parallel wells of white non-phosphorescent 96-well microplate containing 100 μl PBS with (A) or without (B) mixture of 200 μM UTP and 5 U/ml of NDP kinase from baker's yeast *S. cerevisiae* (Sigma). Subsequent to addition of 50 μl ATP-monitoring reagent containing luciferin/luciferase mixture, luminescence of the samples was measured using Tecan Infinite M200 microplate reader (Salzburg, Austria). The differences in luminescence signals between well “A” (ATP + ADP) and “B” (only ATP) allowed quantifying the concentration of ADP, which was converted into ATP through the NDP kinase mediated reaction in the presence of exogenous UTP. Such approach allows simultaneous measurement of both ATP and ADP content within the same sample. Plasma haemoglobin concentration was also determined by measuring the absorbance at the peak of the Soret band (415 nm) and also at 380 and 450 nm, as described previously (Mercier et al., 2012). Hemoglobin levels in all analyzed blood samples did not exceed 4.0 mg/dL (data not shown).

### Measurement of soluble nucleotidase activities

Soluble NTPDase and 5′-nucleotidase activities were assayed radiochemically, as described earlier (Yegutkin et al., 2007). Specifically, for ADPase/NTPDase activity, serum (10 μl) was incubated 60 min at 37°C in 80 μl RPMI-1640 medium containing 5 mM β-glycerophosphate, 80 μM of adenylate kinase inhibitor Ap5A and 50 μM ADP with tracer [2,8-3H]ADP (Perkin Elmer, Boston, USA). Likewise, 5′-nucleotidase activity was assayed by incubating 10 μl serum for 60 min at 37°C in 80 μl RPMI-1640 with 5 mM β-glycerophosphate, 300 μM [2-3H]AMP (Quotient Bioresearch, GE Healthcare, Rushden, UK). Aliquots of the mixture (8 μl; ~5 × 10^5^ dpm/spot) were applied to Alugram SIL G/UV_254_ sheets (Macherey-Nagel, Duren, Germany) and separated by thin-layer chromatography using appropriate solvent mixture (Yegutkin and Burnstock, 1998). Radiolabeled substrates and their dephosphorylated products were quantified by scintillation β-counting and nucleotidase activities were expressed as nanomoles of ^3^H-substrate metabolized per hour by 1 ml serum.

Physiological variability of circulating nucleotide levels and soluble nucleotidase activities were assessed in three males and three females. Blood samples were collected after overnight fasting week apart three times. Coefficients of variation were 0.47, 0.47, 0.18, and 0.27 for ATP, ADP, NTPDase, and 5′-nucleotidase, respectively.

### Echocardiography protocol

Transthoracic echocardiography studies were performed with Sequoia C 512 ultrasound mainframe (Acuson Inc., Mountain View, California, USA) with a standard 3.5 MHz transducer as previously described (Kiviniemi, 2008). B-mode and color-Doppler mapping were used to identify the distal LAD. The coronary flow velocity was assessed with a pulsed wave Doppler and mean diastolic flow velocities (MDV) were measured.

### Cold pressor test

The studies were performed after overnight fasting. Alcohol and caffeine were prohibited 12 h before the study. On the study morning, the subjects took one-half of their normal long-acting insulin and no short-acting insulin. Baseline values of coronary flow velocity were detected as an average of three separate flow velocity measurements. After the baseline measurements, the subjects' right hand was placed into ice-cold water for 120 s. During the hand immersion, flow velocity was measured continuously to allow time course sampling of MDV values as previously described (Kiviniemi et al., 2007). The hyperemia/baseline-ratio of MDV during CPT, the time to the peak MDV during CPT from 0 s time point and the average acceleration slope to the peak were measured as previously described (Dimitrow et al., 2000). The response profiles related to time to peak MDV and acceleration slope to the peak were based on mathematical imputation using non-parametric regression which can give reliable results for reasonable amount of available observations. Profiles with less than 20% data points available throughout the CPT have been discarded to improve the quality of findings. Therefore, adequate coronary flow velocity tracings could be obtained in 14 and 17 patients in pravastatin and placebo groups, respectively. This was due to patients were moving during cold pressor test (CPT) hand immersion.

CPT was generally well tolerated. Intra and inter-observer variabilities of the CPT measurement (hyperemia to baseline ratio) were coefficient of variation (CV) 3.0 ± 2.0%, and 3.7 ± 3.7% respectively, and mean difference 0.0 (−0.1 to 0.1) and 0.0 (−0.2 to 0.2), respectively.

### Statistical analyses

Results are presented as mean value ± SD unless stated otherwise. Primary prospectively determined endpoint of CPT response included hyperemia to baseline ratio, and co-primary end points the time to the peak MDV during CPT from 0 s time point, and the average acceleration slope to the peak (Dimitrow et al., 2000). Sample size of 16 subjects was calculated for each group with a known SD of 0.5 for CPT hyperemia to baseline ratio and an assumed difference of 0.5 between the interventions (α = 0.05, β = 0.80). ATP, ADP levels, and soluble NTPDase and 5′-nucleotidase activity determinations and their association with CPT values were assessed on a *post hoc* basis. Comparisons between the groups were analyzed using the non-parametric Mann–Whitney *U*-test, sign test and Wilcoxon signed-rank tests, or using the parametric unpaired Student's *t*-test where appropriate. For comparison between pre- and post-intervention levels in the same group, paired *t*-test was used where appropriate. Associations between the study variables were assessed using the Pearson's correlation coefficient. A *p*-value of <0.05 was interpreted as statistically significant. Statistical analyses were performed using SAS statistical program package (SAS Institute, Cary, NC, USA) and Matlab (The MathWorks, Inc., Natick, MA, USA).

## Results

### Subject characteristics

The analysis is based on the data from 42 diabetic patients. The baseline characteristics of the study subjects are listed in Tables 1 and 2. There were 13 smokers in the study group (7 and 6 in pravastatin and control groups, respectively). Compliance determined by capsule count exceeded 90%. The study subjects reported no side effects during pravastatin treatment. In one subject receiving pravastatin an asymptomatic rise in serum creatine kinase level was detected at the follow-up visit. The level returned to normal within 2 weeks after cessation of medication. As a marker of glycemic control GHbA_1c_ levels remained unchanged during the intervention in pravastatin and placebo groups.

**Table 1:** **Baseline characteristics of the study subjects and healthy control subjects**.

	**Pravastatin group**	**Placebo group**	**Healthy controls**
*N*	22	20	41
Age (years)	30.2 ± 5.6	28.9 ± 6.5	24 ± 2.3
Males/females	13/9	11/9	41/0
Diabetes duration (years)	13.2 ± 7.8	10.5 ± 5.3	0
BMI (kg/m^2^)	24.7 ± 2.5	24.6 ± 2.6	24.2 ± 2.8
FMD (%)	3.4 ± 3.1	4.9 ± 3.1	N/A
Family history of CAD	4/22	2/20	N/A
Current smokers	7/22	6/20	0/41
Background retinopathy	5/22	3/20	N/A
Autonomic neuropathy	1/22	2/20	N/A
U-Albumin/creatinine	3/22	0/20	N/A
male > 2.5 mg/mmol			
female > 3.5 mg/mmol			

FMD, flow-mediated vasodilation of brachial artery; CAD, coronary artery disease; N/A, data not available.

**Table 2:** **Glycemic control and lipid profile of the study subjects before and after treatment, and healthy control subjects**.

	**Pravastatin (*n* = 22)**		**Placebo (*n* = 20)**		**Healthy control**
	**Baseline**	**Follow-up**	**Baseline**	**Follow-up**	**(*n* = 41)**
HbA_1c_ (%)	8.3 ± 1.1	8.4 ± 1.2	8.2 ± 1.1	8.0 ± 1.1	N/A^*^
Total cholesterol (mmol/L)	4.4 ± 0.5	3.3 ± 0.4^**^	4.6 ± 0.8	4.3 ± 0.6	4.2 ± 0.7
LDL cholesterol (mmol/L)	2.4 ± 0.5	1.6 ± 0.4^**^	2.5 ± 0.6	2.4 ± 0.6	2.3 ± 0.6
HDL cholesterol (mmol/L)	1.6 ± 0.3	1.5 ± 0.3	1.5 ± 0.3	1.5 ± 0.3	1.5 ± 0.4
Triglycerides (mmol/L)	0.8 ± 0.3	0.6 ± 0.2	1.3 ± 1.0	0.9 ± 0.5	0.9 ± 0.4

N/A = data not available.

* Reference range in non-diabetic subjects 4.2–6.0%

** p < 0.001.

### Effect of pravastatin treatment on circulating nucleotide levels and soluble nucleotidase activities

**Figure 1:**
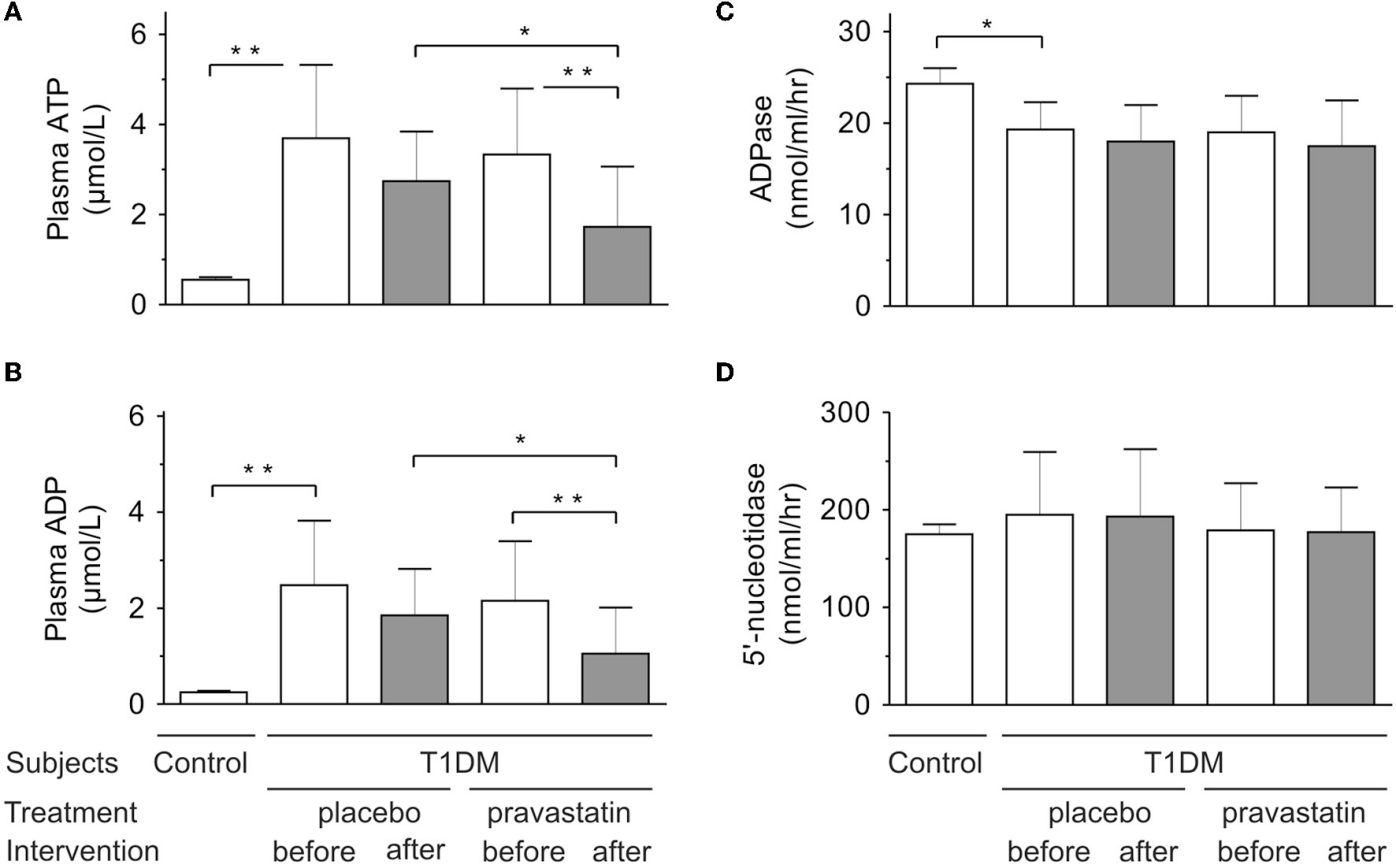
**Effect of pravastatin treatment on circulating nucleotide ATP (A) and ADP (B) levels and soluble nucleotidase ADPase (C) and 5′-nucleotidase activities (D) in type 1 diabetic patients (pravastatin *n* = 22, placebo *n* = 20)**. Control refers to healthy subjects (*n* = 41). ^*^p < 0.05; ^**^p < 0.01.

As shown in Figure 1, there were no significant differences in ATP (panel **A**) and ADP (panel **B**) concentrations between pravastatin and placebo groups before intervention. Noteworthy, both nucleotides were maintained at significantly higher levels in plasma from diabetic patients, as compared to healthy control group.

After intervention, ATP and ADP levels decreased significantly in pravastatin group compared to pre-intervention (*p* = 0.002 and *p* = 0.007, respectively), but not in the placebo group (*p* = 0.06 and *p* = 0.14, respectively). Post-intervention, the ATP and ADP levels were also significantly lower in pravastatin group compared to the placebo group (*p* = 0.02 and *p* = 0.03, respectively), while there was no difference during pre-intervention (*p* = 0.55 and *p* = 0.50, respectively). No signs of haemolysis or differences in plasma hemoglobin were detected among the studied groups.

Since extracellular nucleotide levels generally represent a net balance between nucleotide release and inactivation, we also determined, whether the activities of purinergic enzymes are concurrently shifted in the bloodstream of the diabetic patients. Since correct determination of serum ATPase is complicated by co-existence of two different enzymes possessing nucleotide hydrolase and pyrophosphatase activities (Yegutkin et al., 2007), soluble NTPDase was assayed in the presence of specific adenylate kinase inhibitor Ap_5_A by using [^3^H]ADP as another preferred substrate. The results unambiguously showed that the activities of serum NTPDase (Figure 1C) as well as another nucleotide-inactivating enzyme 5′-nucleotidase (Figure 1D) did not change in either group. Interestingly, the rate of [^3^H]ADP, but not [^3^H]AMP, hydrolysis by serum from diabetic patients was significantly lower in comparison with control group (Figures 1C,D).

### Coronary flow velocity response to cold pressor test

Adequate coronary flow velocity profiles were available for 14 and 17 patients in pravastatin and placebo groups, respectively. Baseline flow velocity, hyperemia/baseline-ratio of flow velocity during CPT and time to the peak flow velocity during CPT are presented in Table 3. The acceleration to the peak flow velocity value increased in the pravastatin group from pre- to post-intervention (*p* = 0.02), but not in the placebo group (*p* = 0.15). It was also marginally different (*p* = 0.05) between the pravastatin and placebo groups post-intervention, but not at baseline (*p* = 0.9).

**Table 3:** **Coronary flow velocity response to cold pressor test (CPT)**.

	**Pravastatin**		***p***	**Placebo**		***p***	***p***	**Healthy controls**	
	**Pre-intervention**	**Post-intervention**	**Pre vs. post**	**Pre-intervention**	**Post-intervention**	**Pre vs. post**	**Pravastatin vs Placebo post**		**Control vs. pre-intervention T1DM**
Baseline flow velocity (m/s)	0.23 ± 0.06	0.25 ± 0.07	0.29	0.22 ± 0.08	0.21 ± 0.06	0.31	0.08	0.22 ± 0.05	0.78
CPT flow velocity (m/s)	0.33 ± 0.08	0.37 ± 0.14	0.22	0.31 ± 0.10	0.30 ± 0.10	0.61	0.11	0.41 ± 0.13	0.002^*^
CPT flow/baseline flow (% increase)	46 ± 24	51 ± 39	0.19	45 ± 46	49 ± 51	0.94	0.93	86 ± 45	<0.001^*^
Acceleration slope to the peak (1 /s)	0.0074 ± 0.008	0.0147 ± 0.016	0.02^*^	0.0069 ± 0.011	0.0047 ± 0.005	0.15	0.05^*^	0.0084 ± 0.0054	0.06
Time to peak flow (s)	58 ± 22	50 ± 21	0.18	55 ± 18	69 ± 25	0.16	0.02^*^	39 ± 21	0.002^*^

Baseline flow, maximal flow during CPT, the percent increase of flow during CPT, acceleration slope to peak flow velocity during CPT and the time to the peak mean diastolic velocity during CPT. The average acceleration (slope) to the peak = hyperemia to baseline value/time to attain the peak

* p ≤ 0.05.

**Figure 2:**
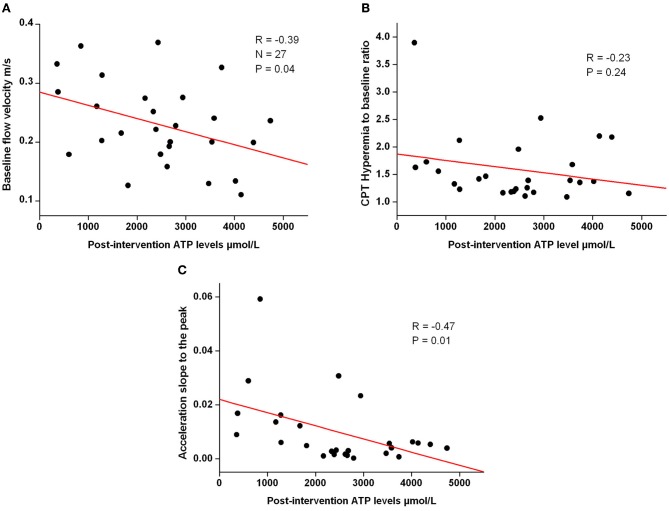
**Figure shows correlation graph of post-intervention baseline coronary flow velocity (**A**) (*n* = 27), CPT flow velocity/baseline flow velocity (**B**) (*n* = 26) and acceleration to the peak slope (**C**) (*n* = 26) vs**. **post-intervention ATP value**.

Baseline flow velocity and the acceleration to the peak flow velocity values were associated with post-intervention ATP levels but hyperemia to baseline ratio was not (Figure 2, panel **A–C**). No association was seen with corresponding values and the ADP levels.

## Discussion

The novel findings of this double blinded prospective controlled trial in patients with T1DM are that, in comparison with placebo group, four-month pravastatin treatment is accompanied by (1) significantly decreased levels of intravascular ATP and ADP without any shifts in soluble nucleotidase activities; and (2) slightly improved coronary flow responses to CPT. Moreover, T1DM patients had significantly higher plasma nucleotide concentrations in comparison with healthy controls. The obtained data on correlation between the elevated plasma ATP and ADP levels and impaired CPT might suggest the implication of purinergic signaling mechanisms in beneficial pleiotropic effects of statins in T1DM patients.

Striking novelty of this study is that, compared to healthy subjects, patients with T1DM had higher circulating ATP and ADP levels at the fasting state and this increment can be partially restored after 4-month treatment with pravastatin. The exact mechanisms underlying the elevated pre-intervention nucleotide levels in T1DM patients remain unknown and might be particularly defined by enhanced ATP release from endothelial cells in response to chronic hyperglycemia (Parodi et al., 2002); increased shear stress due to disturbed blood flow (Yegutkin et al., 2000, 2007); and/or by diminished activity of vascular endothelial NTPDase/CD39 (Robson et al., 2005). In line with the altered nucleotide turnover results, we recently provided evidence of increased levels of circulating ATP and ADP as well as impaired coronary vasodilation response in young pre-atherosclerotic apolipoprotein E-deficient mice versus wild type littermates (Mercier et al., 2012). An increase in glucose from 5 to 15 mmol/l results in a marked increase in the proatherogenic nuclear factor of activated T cells signaling pathway in VSMC (Nilsson et al., 2006). The effect is mediated via glucose-induced release of ATP and UTP, which subsequently activate P2Y_2_ but also P2Y_6_ receptors after degradation to UDP. Thus, nucleotide release is a potential metabolic sensor for the arterial smooth muscle response to high glucose. (Erlinge and Burnstock, 2008) In this context, it is relevant to emphasize that serum NTPDase activities were lower in diabetics, as compared to those of the healthy control group. Moreover, while no significant shifts in soluble nucleotidase activities were detected in the sera from pravastatin-treated T1DM patients versus placebo group, we do not exclude the possibility of statin-mediated up-regulation of vascular nucleotidase activities, which in turn would affect the levels of circulating nucleotides and tune their signaling responses in the cardiovascular system. This hypothesis is indirectly supported by previous *in vitro* data on the ability of various statins (lovastatin, atorvastatin) to increase the activities of ecto-5′-nucleotidase (Ledoux et al., 2002; Sanada et al., 2004; Osman et al., 2006) and other nucleotide-inactivating enzymes (Osman et al., 2006) in vascular endothelial and other cells. Irrespective of the underlying mechanisms, the increment of plasma ATP and ADP in T1DM patients and their decrease after pravastatin treatment would reasonably suggest that a net balance between nucleotide release and inactivation is disturbed in diabetes and it can be partially restored by statins.

This is the first study assessing the role of purinergic signaling molecules in the early stages of coronary microcirculatory dysfunction of T1DM patients without any clinical signs or symptoms of macrovascular disease or microvascular complications. Due to small sample size, the physiological responses to CPT need to be interpreted cautiously. The primary end point—hyperemia to baseline ratio—was not significantly altered, and there was only a slight statistically non-significant increase in baseline flow velocity after pravastatin treatment in T1DM patients. The co-primary end point (acceleration slope to the peak and time to peak flow) differences were more obvious though subtle between the pravastatin and placebo groups. Compared to healthy men, pre-intervention hyperemic flow response was lower in T1DM patients in terms of lower peak flow velocity and longer acceleration time to peak.

We intentionally selected subjects with normal cholesterol levels and good to satisfactory glycemic control who are likely to be representative of the majority of young patients with type 1 diabetes not generally receiving lipid-lowering therapy although being in clearly increased risk in developing CAD. At the time of the recruitment the study subjects had no diagnoses of diabetic complications other than background retinopathy and microalbuminuria in a small number of the subjects. Autonomic neuropathy in type 1 diabetes has been suggested to cause hyper-reactivity to nitro vasodilators in the forearm vasculature (Mäkimattila et al., 1997). Therefore we did not include subjects with FMD of the brachial artery more than 10% in the study. Pravastatin treatment was generally well tolerated and did not worsen glycemic control. Despite the physiological variability of enzymatic activities/nucleotide concentrations for each individual was relatively high (within 18–47%), there was a distinct difference in ATP and ADP levels between T1DM patients and the control group pre-intervention. Moreover, the decrease in ATP levels after intervention was greater than the physiological variability.

Some limitations need to be addressed. Firstly, caffeine—a competitive adenosine receptor antagonist—was withdrawn 12 h before blood sample collection, and CPT and FMD tests. It is possible that some of its effect persisted at the time of testing. Secondly, we measured soluble ADPase and 5′-nucleotidase activities, but also other methods of nucleotidase activity assessment have been reported. In a recent report, the effect of rosuvastatin treatment on ecto-5′-nucleotidase activity on the surface of human mononuclear cells was measured and its up-regulation further correlated with vasodilatory responses to ischemia (Meijer et al., 2010). Nevertheless, this rather heterogenous cell population represents only certain fraction of circulating hematopoietic cells and express relatively low ecto-5′-nucleotidase activity as compared to vascular endothelial cells (Yegutkin et al., 2002), with the latter cells being generally considered the major regulators of intravascular purinergic signaling and hyperaemic responses. From this point of view, we believe that soluble serum 5′-nucleotidase—in conjunction with another key inactivating enzyme NTPDase1/CD39—represents more integrative parameter of systemic shifts in the enzyme levels and might open up further research to assess the potential diagnostic application of purine-converting enzymes in clinical biochemistry.

## Conclusions

Intravascular nucleotide turnover is altered in the early stages of atherosclerosis in T1DM patients as compared to healthy control subjects. Four-month treatment with pravastatin in young normocholesterolemic patients with non-complicated type 1 diabetes decreased circulating ATP and ADP levels and concurrently improved coronary microvascular reactivity to CPT as compared to placebo. The existence of correlation between intravascular ATP and ADP and coronary reactivity also indicates on the physiological relevance of the measured systemic nucleotide levels and may open up further research for future therapeutic or diagnostic applications of purinergic signaling pathways in the treatment of diabetes and other diseases.

### Conflict of interest statement

The authors declare that the research was conducted in the absence of any commercial or financial relationships that could be construed as a potential conflict of interest.

## References

[B1] Bours M. J. L. Swennen E. L. R. Di Virgilio F. Cronstein B. N. Dagnelie P. C. (2006). Adenosine 5′-triphosphate and adenosine as endogenous signaling molecules in immunity and inflammation. Pharmacol. Ther. 112, 358–404 10.1016/j.pharmthera.2005.04.013 16784779

[B2] Dimitrow P. P. Krzanowski M. Nizankowski R. Szczeklik A. Dubiel J. S. (2000). Comparison of the effect of verapamil and propranolol on response of coronary vasomotion to cold pressor test in symptomatic patients with hypertrophic cardiomyopathy. Cardiovasc. Drug Ther. 14, 643–650 10.1023/A:1007871032421 11300365

[B3] Eltzschig H. K. Ibla J. C. Furuta G. T. Leonard M. O. Jacobson K. A. Enjyoji K. Robson S. C. Colgan S. P. (2003). Coordinated adenine nucleotide phosphohydrolysis and nucleoside signaling in posthypoxic endothelium. J. Exp. Med. 198, 783–796 10.1084/jem.20030891 12939345 PMC2194189

[B4] Erlinge D. Burnstock G. (2008). P2 receptors in cardiovascular regulation and disease. Purinergic Signal. 4, 1–20 10.1007/s11302-007-9078-7 18368530 PMC2245998

[B5] Janatuinen T. Knuuti J. Toikka J. Ahotupa M. Nuutila P. Rönnemaa T. Raitakari O. (2004). Effect of pravastatin on low-density lipoprotein oxidation and myocardial perfusion in young adults with type 1 diabetes. Arterioscler. Thromb. Vasc. Biol. 24, 1303–1308 10.1161/01.ATV.0000132409.87124.60 15142864

[B6] Järvisalo M. J. Raitakari M. Toikka J. O. Putto-Laurila A. Rontu R. Laine S. Lehtimäki T. Rönnemaa T. Viikari J. Raitakari O. T. (2004). Endothelial dysfunction and increased arterial intima-media thickness in children with type 1 diabetes. Circulation 109, 1750–1755 10.1161/01.CIR.0000124725.46165.2C 15023875

[B7] Kiviniemi T. O. Toikka J. O. Koskenvuo J. W. Saraste A. Saraste M. Pärkkä J. P. Raitakari O. T. Hartiala J. J. (2007). Vasodilation of epicardial coronary artery can be measured with transthoracic echocardiography. Ultrasound Med. Biol. 33, 362–370 10.1016/j.ultrasmedbio.2006.08.012 17188799

[B8] Kiviniemi T. (2008). Assessment of coronary blood flow and the reactivity of the microcirculation non-invasively with transthoracic echocardiography. Clin. Physiol. Funct. Imaging 28, 145–155 10.1111/j.1475-097X.2008.00794.x 18312446

[B9] Laufs U. La Fata V. Plutzky J. Liao J. (1998). Upregulation of endothelial nitric oxide synthase by HMG CoA reductase inhibitors. Circulation 97, 1129–1135 10.1161/01.CIR.97.12.1129 9537338

[B10] Ledoux S. Laouari D. Essig M. Runembert I. Trugnan G. Michel J. B. Friedlander G. (2002). Lovastatin enhances ecto-5′-nucleotidase activity and cell surface expression in endothelial cells: implication of Rho-family GTPases. Circ. Res. 90, 420–427 10.1161/hh0402.105668 11884371

[B11] Mäkimattila S. Mäntysaari M. Groop P. Summanen P. Virkamäki A. Schlenzka A. Fagerudd J. Yki-Järvinen H. (1997). Hyperreactivity to nitrovasodilators in forearm vasculature is related to autonomic dysfunction in insulin-dependent diabetes mellitus. Circulation 95, 618–625 10.1161/01.CIR.95.3.618 9024149

[B12] Meijer P. Wouters C. W. van den Broek P. H. H. de Rooij M. Scheffer G. J. Smits P Rongen G. A. (2010). Upregulation of ecto-5′-nucleotidase by rosuvastatin increases the vasodilator response to ischemia. Hypertension 56, 722–727 10.1161/HYPERTENSIONAHA.110.155689 20679180

[B13] Mercier N. Kiviniemi T. O. Saraste A. Miiluniemi M. Silvola J. Jalkanen S. Yegutkin G. G. (2012). Impaired ATP-induced coronary blood flow and diminished aortic NTPDase activity precede lesion formation in apolipoprotein E-Deficient mice. Am. J. Pathol. 180, 419–428 10.1016/j.ajpath.2011.10.002 22074736

[B14] Merla R. Daher I. N. Ye Y. Uretsky B. F. Birnbaum Y. (2007). Pretreatment with statins may reduce cardiovascular morbidity and mortality after elective surgery and percutaneous coronary intervention: clinical evidence and possible underlying mechanisms. Am. Heart J. 154, 391–402 10.1016/j.ahj.2007.04.029 17643594

[B15] Nilsson J. Nilsson L. M. Chen Y. Molkentin J. D. Erlinge D Gomez M. F. (2006). High glucose activates nuclear factor of activated T cells in native vascular smooth muscle. Arterioscler. Thromb. Vasc. Biol. 26, 794–800 10.1161/01.ATV.0000209513.00765.13 16469950

[B16] Osman L. Chester A. H. Amrani M. Yacoub M. H. Smolenski R. T. (2006). A novel role of extracellular nucleotides in valve calcification: a potential target for atorvastatin. Circulation 114, I566–I572 10.1161/CIRCULATIONAHA.105.001214 16820639

[B17] Parodi J. Flores C. Aguayo C. Rudolph M. I. Casanello P. Sobrevia L. (2002). Inhibition of nitrobenzylthioinosine-sensitive adenosine transport by elevated D-glucose involves activation of P2Y2 purinoceptors in human umbilical vein endothelial cells. Circ. Res. 90, 570–577 10.1161/01.RES.0000012582.11979.8B 11909821

[B18] Pitkänen O. P. Nuutila P. Raitakari O. T. Rönnemaa T. Koskinen P. J. Iida H. Lehtimäki T. J. Laine H. K. Takala T. Viikari J. S. Knuuti J. (1998). Coronary flow reserve is reduced in young men with IDDM. Diabetes 47, 248–254 9519721 10.2337/diab.47.2.248

[B19] Ralevic V. Burnstock G. (1998). Receptors for purines and pyrimidines. Pharmacol. Rev. 50, 413–492 9755289

[B20] Robson S. C. Wu Y. Sun X. Knosalla C. Dwyer K. Enjyoji K. (2005). Ectonucleotidases of CD39 family modulate vascular inflammation and thrombosis in transplantation. Semin. Thromb. Hemost. 31, 217–233 10.1055/s-2005-869527 15852225

[B21] Sanada S. Asanuma H. Minamino T. Node K. Takashima S. Okuda H. Shinozaki Y. Ogai A. Fujita M. Hirata A. Kim J. Asano Y. Mori H. Tomoike H. Kitamura S. Hori M. Kitakaze M. (2004). Optimal windows of statin use for immediate infarct limitation: 5′-nucleotidase as another downstream molecule of phosphatidylinositol 3-kinase. Circulation 110, 2143–2149 10.1161/01.CIR.0000143830.59419.73 15451788

[B22] Yegutkin G. G. Burnstock G. (1998). Steady-state binding of [3H]ATP to rat liver plasma membranes and competition by various purinergic agonists and antagonists. Biochim. Biophys. Acta 1373, 227–236 10.1016/S0005-2736(98)00108-4 9733971

[B23] Yegutkin G. G. Henttinen T. Samburski S. S. Spychala J. Jalkanen S. (2002). The evidence for two opposite, ATP-generating and ATP-consuming, extracellular pathways on endothelial and lymphoid cells. Biochem. J. 367, 121–128 10.1042/BJ20020439 12099890 PMC1222875

[B24] Yegutkin G. G. Samburski S. S. Mortensen S. P. Jalkanen S. González-Alonso J. (2007). Intravascular ADP and soluble nucleotidases contribute to acute prothrombotic state during vigorous exercise in humans. J. Physiol. 579, 553–564 10.1113/jphysiol.2006.119453 17204504 PMC2075398

[B25] Yegutkin G. Bodin P. Burnstock G. (2000). Effect of shear stress on the release of soluble ecto-enzymes ATPase and 5?-nucleotidase along with endogenous ATP from vascular endothelial cells. Br. J. Pharmacol. 129, 921–926 10.1038/sj.bjp.0703136 10696091 PMC1571919

